# Stearoyl-CoA desaturase 5 (SCD5) in lipid remodeling: From molecular control to pathophysiology

**DOI:** 10.1016/j.jlr.2026.101078

**Published:** 2026-06-12

**Authors:** Veronika Zámbó, Gabriella Orosz, Miklós Csala, Éva Kereszturi

**Affiliations:** Department of Molecular Biology, Semmelweis University, Budapest, Hungary

**Keywords:** stearoyl-CoA desaturase 5 and fatty acid desaturation, lipid remodeling, transcriptional and post-transcriptional regulation, cancer, metabolic and neurodegenerative diseases, genetic variations

## Abstract

Stearoyl-CoA desaturase (SCD) forms double bonds at the *Δ*9 position of saturated acyl-CoAs, primarily palmitoyl-CoA, and stearoyl-CoA. Of the two human isoforms, SCD1 is ubiquitous, while the tissue-specific SCD5 is expressed in the brain, pancreas, and reproductive tissues, where it facilitates local lipid remodeling and modulates cellular signaling in a context-dependent manner. Its activity significantly impacts membrane composition, vesicular trafficking, and post-translational processing of Wnt proteins, thereby influencing cell fate and signal transduction pathways like EGFR and Akt. The multifaceted regulation of SCD5 involves transcriptional activation by SREBP1 and EGR2, as well as repression by HIF2*α* and NME2. Post-transcriptional modulation includes alternative splicing, which yields SCD5A and SCD5B variants, and the inhibitory actions of specific microRNAs, such as miR-221/222 and miR-145-5p. Notably, the SCD5 protein lacks the N-terminal PEST degradation motifs characteristic of SCD1, which contributes to its distinct stability profile across different cell types. SCD5 plays context-dependent roles in various human cancers. While its downregulation in melanoma and breast cancer is associated with advanced malignancy and epithelial-to-mesenchymal transition (EMT), its expression in glioblastoma stem-like cells is essential for survival and DNA repair. Beyond cancer, genetic polymorphisms and expression alterations of SCD5 have been linked to neurodegenerative diseases, psychiatric conditions like schizophrenia, and metabolic dysfunctions such as visceral adiposity and type 2 diabetes. Understanding these tissue-specific regulatory milieus is crucial for developing SCD5-targeted therapeutic strategies. Here, we review available knowledge regarding the structure, regulation, and function of SCD5, with focus on its role in cell signaling and disease.

Lipids are fundamental components of cellular structure and function, contributing to membrane organization, energy storage, and signal transduction. Fatty acids (FAs), the obligatory building blocks of compound lipids, play a central role as modulators of biophysical properties of the membranes and precursors of various bioactive molecules. Membrane lipid composition, particularly the relative abundance of saturated FAs (SFAs), monounsaturated FAs (MUFAs), and polyunsaturated FAs (PUFAs) determines membrane fluidity, curvature, and the activity of membrane-associated proteins (1). While SFAs tend to promote tighter lipid packing and reduced membrane fluidity, unsaturated FAs introduce structural disorder that enhances membrane flexibility, which affects processes such as vesicular trafficking and signal transduction (2, 3). In addition, PUFAs mobilized from membrane lipids serve as precursors for eicosanoids with diverse biological functions (4). These lipid mediators play an important role, among others, in the control of immune responses and inflammation, as well as cell proliferation and differentiation (5). Together, these diverse functions clearly highlight the importance of maintaining a balanced FA composition in cellular lipids.

The content of FAs ingested with food depends largely on dietary habits. Excessive consumption of SFAs poses health risks, including lipotoxicity and elevated LDL cholesterol levels (6). A diet abundant in saturated fats promotes obesity, insulin resistance and an increased risk of cardiovascular disease, whereas consumption of cis-unsaturated fats generally improves lipid profiles and heart health (7, 8). Naturally occurring cis-unsaturated FAs, e.g., oleate and linoleate, confer metabolic benefits. However, dietary trans FAs are not necessarily healthy, as industrial trans fats, containing trans-elaidate raise LDL and lower HDL cholesterol, thereby promoting atherogenesis and inflammation (7, 8, 9). Therefore, current guidelines recommend minimizing both saturated and trans-fat intake (7).

A key mechanism controlling FA composition is enzymatic desaturation, which introduces double bonds into saturated acyl chains. The first and rate-limiting step of FA desaturation is catalyzed by stearoyl-CoA desaturases (SCDs), endoplasmic reticulum-localized enzymes that form the initial cis-double bond at the *Δ*9 position of long-chain saturated acyl-CoAs. In mammals, SCDs convert palmitoyl-CoA (C16:0) and stearoyl-CoA (C18:0) into palmitoleoyl-CoA (C16:1n-7) and oleoyl-CoA (C18:1n-9), respectively (10, 11). Humans express two SCD isoforms, the ubiquitous SCD1 and the tissue-restricted SCD5 (12). Although SCD5 can also desaturase C18:0 and produce oleate (13, 14, 15, 16, 17, 18, 19, 20, 21, 22), its substrate selectivity may differ slightly from SCD1, with a possible preference for C16:0 desaturation (23) that still needs further confirmation. SCD1-deficient mice exhibit resistance to diet-induced obesity (24, 25) and hepatic steatosis (26, 27), evidencing its pivotal role in lipogenesis. SCD1 activity has been identified as a key determinant of the cellular SFA/MUFA ratio (28, 29, 30). High SCD1 levels promote proliferation and survival by supplying MUFAs for membrane biogenesis and signaling (31, 32). In tumors, increased SCD1 expression is associated with aggressiveness and poor prognosis (33, 34, 35, 36, 37). By regulating membrane fluidity and oncogenic signaling pathways, SCD1 acts as a key metabolic hub (31, 32, 37, 38) and a potential therapeutic target in obesity, diabetes, and cancer (39, 40, 41).

Significantly less is known about SCD5, a second human *Δ*9-desaturase with a more restricted and tissue-specific expression pattern, primarily in the brain, pancreas, and certain reproductive tissues (12, 42). The *SCD5* gene (Fig. 1A) is located on chromosome 4 (43), and the protein shows approximately 55%–58% identity with the amino acid sequences of other SCDs (12). Its orthologs have been identified in primates and several other vertebrates (including ruminants, pigs, dogs, and birds), but not in rodents. Phylogenetic analyses indicate that the emergence of separate *SCD1* and *SCD5* lineages resulted from an early vertebrate gene duplication event. This evolutionary divergence then led to separate expression patterns (12, 44, 45, 46, 47). Two transcript variants (NM_001037582.3 and NM_024906.3), which differ in their last exons, encode SCD5A and B proteins of 330 and 256 amino acids, respectively, whose C-terminal amino acid sequences differ (48) (Fig. 1B). The SCD5 protein contains the canonical four transmembrane helices and three conserved histidine-box motifs (H-X-H) of *Δ*9-desaturases (49, 50) (Fig. 1C). Unlike SCD1, SCD5 lacks PEST degrons in its N-terminus, which may reasonably cause a difference in the stability of the two proteins (44).

**Fig. 1:**
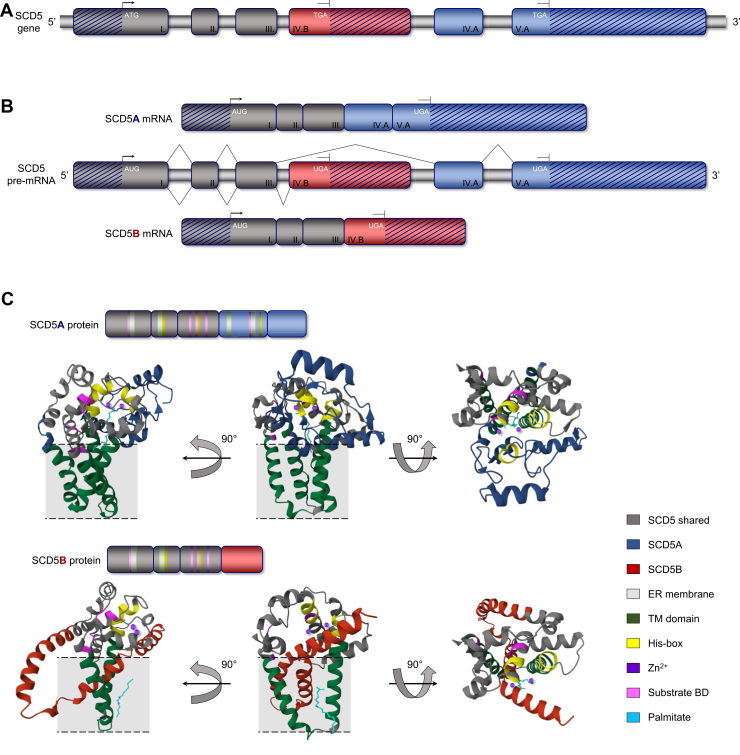
Structure of SCD5 transcript variants. In the schematic representation of the structure of the SCD5 gene (A), pre-RNA, mRNA (B), and protein (C), the shared region of the two transcript variants is shown in gray. The region specific to SCD5A is marked in blue, and the region specific to SCD5B is indicated in red. The crystal structure of the wild type human SCD5A was obtained from the Protein Data Bank (file Q86SK9). The 3D structure of SCD5B was *predicted* and rendered by AlphaFold Server. A previous figure from the present working group has been revised and expanded (48).

Growing evidence suggests that SCD5 does not simply duplicate the function of SCD1 but instead plays specialized roles in local lipid remodeling and in the modulation of signaling pathways. In contrast to SCD1, which has been demonstrated to be highly responsive to diet and hormones (51), SCD5 expression has been found to be relatively insensitive to dietary lipids or serum factors (23, 52, 53, 54). While SCD1 typically supports growth with largely varying effects, SCD5 has been shown to strongly promote neuronal cell proliferation and suppress differentiation (neurite outgrowth). SCD5 is implicated in developmental and neural processes and regulates cell fate by influencing lipid synthesis and signaling pathways (23, 55, 56). However, the precise molecular mechanisms underlying these effects, as well as the physiological significance of SCD5-specific lipid remodeling, remain incompletely understood.

Alterations in SCD5 expression have been associated with a wide range of pathological conditions, including cancer, metabolic dysregulation, and neurological disorders, often in a context-dependent manner. In this review, we summarize current knowledge on the structure, regulation, and function of SCD5, with particular emphasis on its role in cellular signaling and disease. We also highlight key knowledge gaps and discuss future directions required to clarify the biological and therapeutic relevance of this underexplored enzyme of lipid metabolism.

## Regulation of SCD5 Expression

### Transcriptional regulation of SCD5

#### Expression in different tissues

The tissue expression profile of the SCD5 isoform differs substantially from that of the more ubiquitous SCD1 and exhibits pronounced interspecies variability (12, 44, 57). While initially considered unique to primates, subsequent research has confirmed its presence in several other vertebrates, including cattle, pigs, sheep, chickens, and zebrafish (44, 57, 58).

In humans, *SCD5* expression is highly tissue-specific, with the highest levels detected in the brain and pancreas (12, 48, 57, 59). It is abundant in both adult and fetal brain tissue, and expression is higher in the fetal than in the adult brain, suggesting a possible role in neurodevelopment (12, 59). Within the brain, no significant difference in expression has been observed between gray and white matter (57). Recent studies have identified SCD5 as a key contributor to glioblastoma stem cell (GSC) maintenance. In these tumor cells, SCD5 protein is uniformly and strongly expressed, whereas SCD1 shows a more heterogeneous distribution. Notably, SCD5 expression is lost upon GSC differentiation, indicating a specific requirement for stem-cell-state preservation (22).

Substantial amounts of the enzyme are also present in the gonads and cumulus cells, where expression increases during oocyte maturation (48, 59, 60). Lower, but still detectable, levels are found in the kidney, lung, and adipose tissue (12, 48, 59). In humans, *SCD5A* remains the predominant transcript variant, whereas *SCD5B* appears to have minimal biological relevance in glioma models (22).

In cattle, the expression pattern broadly resembles that seen in humans, with the highest mRNA levels reported in the brain (44, 57). Elevated expression is also observed in the kidney and spleen (61). By contrast, only low levels are detectable in the liver, lung, ileum, and colon, and expression in skeletal muscle is very limited (57, 61). In Fad3 transgenic cattle, *SCD5* is upregulated in fibroblasts, promoting fatty-acid desaturation (61). Among other livestock species, such as pigs and sheep, the highest *SCD5* mRNA expression is likewise found in the brain (44). In chickens, however, the transcript is most abundant in both the pancreas and the brain (44). In zebrafish, *SCD5* expression is strongest in the brain, followed by the pancreas and visceral adipose tissue; all three tissues exhibit markedly higher levels than the colon, subcutaneous adipose tissue, spleen, muscle, heart, or liver (58).

#### Promoter characterization

Since the initial description of SCD5 in 2005 (12), it has required approximately two decades to thoroughly characterize its 5′ regulatory region (59). Five sequences of varying lengths, located upstream of the start codon, were examined in a luciferase reporter system in cell lines of neural, liver, and kidney origin. All reporter constructs tested displayed significantly higher activity in neuroblastoma cell lines than in liver- or kidney-derived cells, which is consistent with the previously described tissue specificity of *SCD5* mRNA expression (59). This finding suggests the presence of a cell type-specific regulatory mechanism, the exact nature of which remains to be elucidated. However, in contrast to *SCD1*, whose promoter activity is typically strongly influenced by saturated and cis-unsaturated FAs, i.e., its substrates and products, as well as trans-unsaturated FAs (51), the *SCD5* promoter has been shown to be insensitive to a wide range of FAs (oleate, palmitate, stearate, linoleate, vaccenate, and elaidate) covering these three categories (59). The functionality of a repressor sequence, yet to be fully characterized, is suspected in the sequence between nucleotides −505 and −206 in the 5′ regulatory region of bovine *SCD5* (62), and this is partially detectable in the human promoter as well (59).

#### Transcription factors

Characterization of the *SCD5* promoter was driven by a need to understand the transcriptional regulation of the gene. Although extensive research has been devoted to the control of *SCD1* gene expression, the influencing hormones and nutrients, their mechanisms of action, and the transcription factors involved (51), many questions remain to be answered regarding the regulation of *SCD5* mRNA levels. Due to the identical reaction catalyzed by the two isoforms and based on the known functions and regulatory molecules of SCD1, the initial focus was on the similarities between the promoters of the two desaturases. This approach led to the search for transcription factors (TFs) involved in the regulation of *SCD5* gene expression among those TFs that definitely bind to the *SCD1* promoter. Utilizing in silico tools, eight TFs were identified whose consensus sequence is found in the promoters of both isoforms (46), and they partially overlap with the regulatory proteins predicted in the 5′ regulatory region of *SCD5* in chicken (63). However, their actual binding has yet to be proven. Another study using an analogous method arrived at a different conclusion and identified 15 additional TFs as potential *SCD5* regulatory molecules (59). In a study on epithelial-to-mesenchymal transition (EMT) in melanoma, a correlation was demonstrated between SCD5 expression and EMT-characteristic changes, that is, Zinc Finger E-Box Binding Homeobox 2 (ZEB2) positivity, and ZEB1, Twist Family BHLH Transcription Factor (TWIST), and Snail Family Transcriptional Repressor 2 (SNAI2/SLUG) negativity. Furthermore, SCD5 overexpression was shown to increase the intracellular amount of Microphthalmia-associated transcription factor (MITF), indicating promotion of differentiation (16). Interestingly, administration of an RXR agonist was observed to reduce *SCD5* transcription in neural cell lines and in samples from patients diagnosed with Parkinson’s disease (64). While the entirety of the aforementioned information is of scientific interest, it should be noted that the data merely reflect expression correlations and sequence-analysis results (Supplemental Table S1).

**Fig. 2:**
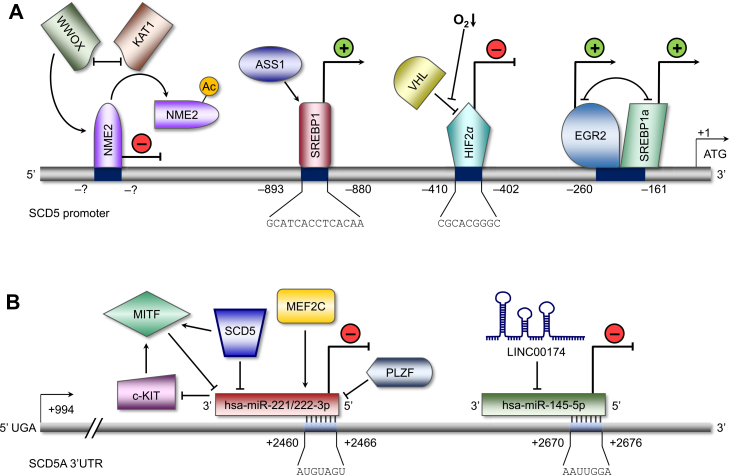
Regulatory elements located in the 5′UTR (A) and 3′UTR (B) regions of the SCD5 gene. Functional transcription factor binding sites are marked in dark blue, and microRNA consensus sequences are highlighted in light blue; their exact positions and sequences are also provided where available. Activating interactions between regulatory elements are indicated by an arrow, while inhibitory effects are shown by an inhibition arrow. The direction of the effect of regulatory units on SCD5 levels is illustrated by a green-circled + sign or a red-circled - sign. A detailed description of the regulatory mechanisms can be found in the text. Ac: acetylation.

The most reliable evidence regarding TFs and regulatory mechanisms comes from functional studies that adequately reflect in vivo conditions. The putative role of four TFs has already been convincingly demonstrated in such models (Fig. 2A). The first functional TF - promoter interaction was discovered during the investigation of the 5′ regulatory region of bovine *SCD5* in an in vitro luciferase reporter system. In rodents, the TFs early growth response 2 (EGR2) and sterol regulatory element binding protein 1a (SREBP1a) regulate transcription of the stearoyl-CoA desaturase 2 (*SCD2*) gene during peripheral nerve myelination, likely playing an important role in the synthesis of the lipid components of myelin (65, 66). The majority of non-rodent genomes lack the *SCD2* gene (45), with the *SCD5* gene being expressed in the brain and neural tissue instead (12, 44, 48, 57, 58, 59, 60, 61). The consensus sequences of EGR2 and SREBP1a were also identified in the *SCD5* promoter in the region between nucleotides −260 and −161. Both TFs individually increased *SCD5* transcription, but due to their binding to the same region of the promoter, they were found to inhibit each other's effects when combined (62). As part of research aimed at developing a potential antitumor strategy, the binding of SREBP1 to the human *SCD5* promoter was confirmed in tumor cells using a dual luciferase reporter system and then further validated by ChIP-qPCR. In this process, ASS1, a pivotal enzyme in the urea cycle that plays a significant role in ferroptosis resistance, is responsible for SREBP1 recruitment and the consequent increase in *SCD5* transcription (67) (Fig. 2A).

The role of hypoxia-inducible factor (HIF) in modulating hypoxic responses in tumors through altering cellular energy metabolism is well-documented, as is its impact on the modification of glucose and lipid metabolism-associated gene expression. Its role in enhancing *SCD1* expression in clear cell renal cell carcinoma (ccRCC) has been described previously (68), and the HIF2*α* binding site has also been identified in the *SCD5* promoter (69) (Fig. 2A). However, while HIF2*α* definitely increases *SCD1* expression, it significantly inhibits *SCD5* transcription. The tumor suppressor E3 ubiquitin ligase component, von Hippel-Lindau (VHL), which facilitates HIF2*α* degradation, is often down-regulated in human ccRCCs (70). This leads to a decrease in *SCD5* transcription via HIF2*α* binding in these tumor cells, which is further augmented by low oxygen concentrations (69).

NME2 has also been identified as a repressor of *SCD5* transcription in human hepatocellular carcinoma in a dual-luciferase reporter system and confirmed by a pull-down assay (20). NME2 plays a crucial role in controlling the mechanism of metastasis, and its anti-metastatic function has been implicated in numerous tumor cells (71). The binding of NME2 to the *SCD5* promoter and the resulting inhibition of *SCD5* transcription are influenced by the ratio of two proteins, WWOX and KAT1, which compete for NME2 binding and exert antagonistic effects on this TF (Fig. 2A). WW domain-containing oxidoreductase (WWOX) is TF-binding tumor suppressor protein that modulates many cellular behaviors, including metabolic and tumorigenic processes (72). WWOX forms a complex with NME2, thereby enhancing its DNA binding ability and consequently inhibiting *SCD5* expression. It should be noted that it can also stabilize the binding of HIF to the *SCD5* promoter in a similar manner (73), thereby synergistically enhancing the inhibition of *SCD5* transcription (69) (Fig. 2A). The other important interacting partner of NME2 is KAT1 histone transacetylase, which acetylates lysine 31 of NME2, thus promoting its dissociation from DNA. Therefore, KAT1 can increase *SCD5* transcription by lifting its NME2-mediated repression (72).

### Post-transcriptional and translational regulation

*SCD1* and *SCD5* show a remarkable difference regarding mRNA stability. The half-life of *SCD1* mRNA has been found to be as short as 2–4 h, almost identical to that of the protein (see below) (74). In contrast, *SCD5* mRNA appears to be quite stable, with its quantity halving every 15 h in melanoma cells (16).

MicroRNA (miRNA) is a small, single-stranded, non-coding RNA molecule that plays a key role in regulating gene expression at the post-transcriptional level. Its primary target is the 3′ untranslated region (3′UTR) of selected mRNAs, and its main function is RNA silencing. miRNAs can suppress the production of proteins encoded by target mRNAs by binding to a specific sequence in the 3′UTR and thereby inhibiting translation or even enhancing mRNA degradation (75, 76). Recent research, mostly based on sequence analysis, has suggested that *SCD5* may be targeted by a number of miRNAs (Supplemental Table S2). However, since an imperfect complementarity of six to eight nucleotides in the seed region is sufficient for effective miRNA binding, the accuracy and reliability of predictions is limited, and all in silico results need to be validated under experimental conditions. Furthermore, as discussed above, isoforms A and B of *SCD5* have different 3′UTR sequences due to alternative terminators, so the two *SCD5* variants may interact with diverse miRNAs (see Fig. 1A, B). While no miRNA binding has been described for *SCD5B*, either in silico or in vitro, seven conserved regions of miRNA families were identified by predictions in the 3′UTR of *SCD5A*, the vast majority of which are closely related to various types of cancer (46, 77). Several of the miRNAs predicted to bind to *SCD5A* mRNA are associated with neurological conditions, which is noteworthy given the high expression level of *SCD5* in the brain. For instance, miR-20b is linked to schizophrenia (46, 78, 79), miR-106a to autism (46, 80), miR-17 to glioma (46, 81), and miR-1928 to PTSD (82). It is also remarkable that the binding sites for the miRNAs associated with pancreatic cancer, such as miR-205, miR-221, miR-222, miR-17-5p, and miR-20a, have been identified in the 3′UTR of *SCD5A*, which is known to be highly expressed in the pancreas (83, 84, 85, 86). Furthermore, elevated levels of miR-34b expression have been observed in T-cells from patients diagnosed with rheumatoid arthritis, and the expression of *SCD5*, as a predicted target of miR-34b, has been found to decrease significantly in these cells (87). On the other hand, only a few miRNA binding sites associated with disorders of carbohydrate and lipid metabolism can be found in the 3′UTR of *SCD5A*. Among these, members of the miR-200ab and miR-17 families are associated with non-alcoholic fatty liver disease (NAFLD) (46, 88, 89), while miR-484, involved in glucose metabolism and insulin resistance, has been implicated in the development of type 2 diabetes mellitus (T2DM) (90, 91, 92).

Currently, only two experimentally validated miRNA binding sites are known in the 3′UTR of *SCD5A* (Fig. 2B). Puglisi *et al.* observed a negative correlation between miRNA miR-221/222 levels and *SCD5* mRNA and protein levels in different primary and metastatic melanomas (16). The miR-221-3p and miR-222-3p consensus binding sites were first identified in silico and then validated using an in vitro luciferase reporter system. The miR-222 binding site in the 3′UTR of pig *SCD5* has been validated in a luciferase reporter system and appears to be highly conserved across species (93). The observation that overexpressed SCD5 reduces miR-221/222 levels strongly suggests a negative feedback relationship between SCD5 and this miRNA. Although it cannot be ruled out that SCD5 directly inhibits miR-221/222 expression, microphthalmia transcription factor (MITF) seems to play an important role in this effect. MITF is a pivotal transcription factor and master regulator for the development and function of melanocytes and retinal pigment epithelial cells. It controls genes involved in cell survival and proliferation, and its dysregulation has been linked to cancers such as melanoma. SCD5 is known to enhance MITF expression, which in turn can downregulate miR-221/222 (Fig. 2B) by targeting its promoter (94). Besides, miR-221/222 also feeds back on its own expression through MITF, as c-KIT is also a target of miR-221/222, and its inhibition reduces the inhibitory effect of MITF (95) (Fig. 2B). Additionally, SCD5 levels in melanoma can also be increased by the TF promyelocytic leukemia zinc finger protein (PLZF), which has been identified as a repressor of the miR-221/222 family (95) (Fig. 2B). It has also been demonstrated, using ChIP and EMSA methods, that the mitogenic TF MEF2C targets and upregulates miR-222, thereby inhibiting *SCD5* expression (93). The other miRNA that has been experimentally shown to bind to the 3′UTR of *SCD5* is miR-145-5p, which plays a key role in suppressing thymic epithelial tumors (96). A long non-coding RNA (lncRNA) LINC00174 can favor the expression of SCD5 by sponging miR-145-5p, and this mechanism has been implicated in promoting the migration of thymic epithelial cancer cells (97) (Fig. 2B).

### Post-translational regulation

The stability of proteins is an important determinant of their actual abundance in the cell. The N-terminal amino acid sequence and post-translational modifications significantly influence protein half-life. The paucity of data on the degradation profile of SCD5 is particularly striking given the extensive characterization of the other human isoform, SCD1. The PEST domain identified and functionally analyzed at the N-terminus of SCD1 accounts for the relatively short, 2–4 h half-life of this desaturase (74, 98). Despite the overall 54%–58% identity and 72%–74% similarity of the amino acid sequences (12, 43), the N-termini of SCD1 and SCD5 are completely different, which is also reflected in the fact that both human (22) and bovine SCD5 (57) lack the PEST domain. Despite differences in PEST motifs, protein stability assays showed comparable degradation rates for both isoforms in primary glioblastoma stem-like cells within eight hours after cycloheximide-induced translational inhibition, though they occupy distinct functional microdomains within the ER (22). However, degradation of SCD5 shows large variations and thus appears to be subject to regulation. Comparing early primary melanoma cells and metastatic melanoma cells representing the initial and advanced stages of the tumor, using cycloheximide treatment, the half-life of the SCD5 protein was found to be 12 h in the former, while only about one-tenth of that, approximately 1.5 h, in the latter (16). Similar to SCD1, the degradation of SCD5 can only be partially prevented by MG132 treatment, suggesting that, in addition to the proteasomal pathway, other degradation routes, such as calpain-mediated proteolysis, play a role in regulating intracellular levels of desaturase proteins (16, 99, 100). Importantly, all data regarding SCD5 relate to the SCD5A isoform, as no experimental information on the stability of SCD5B are available to date. Since SCD5B is undetectable or barely detectable at the protein level in glioblastoma (22), and its mRNA expression is a fraction of that of *SCD5A* in virtually all human tissues (48), this isoform may reasonably be considered biologically irrelevant.

Very little research data is available on post-translational modifications of human desaturases. In the case of human SCD1, it has been demonstrated that EGFR stabilizes the enzyme through phosphorylation of tyrosine 55, thereby upregulating MUFA synthesis (101). The stabilizing effect is partly due to the prevention of polyubiquitination by phosphorylation (99). However, since the N-terminal region, which includes amino acid 55, differs significantly between the two human isoforms, a similar mechanism cannot be reasonably postulated for SCD5. Two large-scale, data-driven studies on SCD5 suggest that lysine 173 and 315 are ubiquitinated in the enzyme protein, but this still awaits experimental confirmation (102, 103).

## Role of SCD5 activity at the cellular level

Experimental data concerning the intracellular effects exerted by the expression, enzyme activity, and products of SCD5, as well as on specific causal relationships, are scarce. While there is compelling evidence that both stearoyl-CoA and palmitoyl-CoA are substrates of SCD1 (29), the assessment of this issue is considerably more uncertain in the context of SCD5. According to the prevailing opinion in scientific publications, SCD5 is predominantly an oleate-producing enzyme (14, 16, 17). The impact of oleate on the body is also being investigated in *SCD5* transgenic mouse models (13, 15, 18, 19), and in certain instances, alterations in SCD5 activity have been established by quantifying oleate levels (20). However, it seems plausible that 16-carbon SFAs are also substrates for SCD5 (12, 21). In fact, overexpression of SCD5 in mouse neural cell lines resulted only in an increase in palmitoleic acid levels, with no change in the concentrations of palmitate, oleate, and stearate (23).

**Fig. 3:**
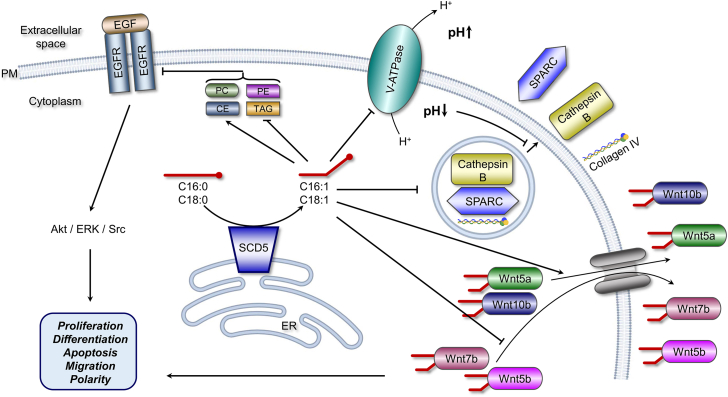
Intracellular consequences of SCD5 enzyme activity. A graphical summary of currently known SCD5-dependent intracellular processes. The direction of the effect of regulatory units on SCD5 levels is illustrated by a green-circled + sign or a red-circled - sign. PM: plasma membrane, PC: phosphatidylcholine, PE: phosphatidylethanolamine, CE: cholesteryl esters, TAG: triacylglycerol.

Constitutive overexpression of SCD5 in neuroblastoma cells resulted in enhanced synthesis of phosphatidylcholine and cholesteryl esters, and reduced formation of phosphatidylethanolamine and triacylglycerol. Presumably, due to the resulting change in plasma membrane composition, epidermal growth factor (EGF)-induced EGFR, Akt and ERK phosphorylation was significantly attenuated in SCD5-expressing cells. Along with marked changes in canonical and non-canonical Wnt signaling (see below), the SCD5-induced modulations reduced neural differentiation and increased proliferation in neuroblastoma cells (23) (Fig. 3). On the other hand, in melanoma-derived cell lines, it has been observed that elevated intracellular SCD5 protein levels promote cell differentiation (16). Moreover, both in cell culture and *C. elegans* models, a correlation was demonstrated between increased proliferation and diminished SCD5 mRNA and protein levels (69). The SCD5 protein is clearly detectable in primary cutaneous melanoma, but the enzyme is virtually absent from the metastases of the tumor. Restoring SCD5 expression significantly reduced melanoma progression both in vitro and in vivo, presumably by inhibiting the secretion of certain extracellular matrix components, such as secreted protein acidic and rich in cysteine (SPARC) and collagen IV and of their proteases, such as cathepsin B (14). Oleate-dependent inhibition of SPARC secretion, leading to reduced metastatic spreading in SCD5 overexpressing cells was also confirmed in a triple-negative breast cancer mouse model (18) (Fig. 3).

Wnt proteins are a family of signaling molecules that regulate essential developmental processes, such as cell proliferation, differentiation and migration, as well as the homeostasis of adult tissues. Aberrant Wnt signaling is linked to a variety of diseases, including cancers, fibrosis, metabolic disorders, and neurodegenerative conditions (104, 105). The activity of SCD5 can significantly influence post-translational modifications that are essential for the optimal secretion and activity of Wnt proteins, as both S-palmitoylation of a cysteine side chain and O-palmitoylation of a serine residue can occur with the use of the corresponding SFA or MUFA (106, 107). Enhanced SCD5 activity has been shown to reduce the enzymatic activity and secretion of canonical Wnt7b, while having the opposite effect on non-canonical Wnt5a (23). Modulation of the Wnt pathways by SCD5 activity is further supported by the fact that, similar to Wnt7b, the level of canonical Wnt5b was also downregulated in SCD5-overexpressing bovine samples (108). In addition, *β*-catenin-dependent Wnt10b expression was shown to be significantly reduced in *SCD5* knockout zebrafish (58) (Fig. 3).

In many cases, mainly in tumor models, positive or negative correlations have been described between SCD5 and certain phenomena or levels of molecules, which have not yet been explained by a proven direct relationship (see Table 1). Nevertheless, these data reveal some key intracellular processes that could help determine the position of the SCD5 enzyme and the reaction it catalyzes across various phases of cell life. Consistent with its significant expression in the gonads and during early embryonic development, SCD5 appears to play a role in oocyte maturation, and blastocyst development, both of which significantly affect embryo quality (60, 109, 110).

**Table 1:** Biological features associated with SCD5 levels

Process Description	Change in the Level of SCD5		Model System	Sample Source	Reference
	Protein	mRNA			
Increased oocyte nucleus maturity		↑	human sample	human cumulus cells	(60)
Declined embryo quality		↑	high-throughput	human cumulus cells	(109)
*In vitro* produced blastocytes		↓	high-throughput	bovine oocytes	(110)
Increased tyrosinase enzyme level	↑		cell culture	A375M	(16)
Increased cell differentiation	↑		cell culture	A375M	(16)
Increased cell proliferation	↓	↓	cell culture, animal model	HEK293T, HeLa, 786-O, RCC4, *C. elegans*	(69)
E-cadherin localization change	↑		cell culture	A375M	(16)
Reduced cell migration	↓		cell culture	TC1889	(97)
Reduced lipid droplet content	↓		cell culture	TC1889	(97)
Reduced PLIN-2 protein level	↓		cell culture	TC1889	(97)
Reduced lipid droplet size		↓	cell culture	SUM159, THP-1	(64, 111)
Increased visceral adipose tissue	↓	↓	animal model	zebrafish	(58)
Nutrient deprivation		↑	high-throughput	scallop hepatopancreas	(113)
Isoxazole treatment		↑	cell cultures	human islets	(112)
Lysosomal translocation of lipids	↑		cell culture, animal model	*C. elegans*, HEK293T	(114)
Increased necrosis	↓	↓	cell culture	MCF-7, MDA-MB-231	(17)
Increased ferroptosis resistance	↑		cell culture	NSCLC, HBE	(67)
Increased ferroptosis sensitivity	↓	↓	cell culture	PC3, HepG2, A549	(19)
Induced parthanatos	↓	↓	cell culture	primary glioblastoma stem-like cells	(22)
Reduced ROS production	↑		cell culture, animal model	*C. elegans*, HEK293T	(114)
Reduced apoptosis	↑		cell culture, animal model	*C. elegans*, HEK293T	(114)

Increased *SCD5* mRNA and protein expression promotes cell differentiation over proliferation in certain tumors (16, 69). Increased SCD5 levels are associated with increased cell-to-cell adhesion and maintenance of epithelial tissue integrity, through stimulation of critical E-cadherin synthesis and induction of its translocation. An increase in the levels of the dedifferentiation marker tyrosinase enzyme has also been observed in melanoma-derived cell lines (16). However, this is somewhat contradicted by the observation that increased SCD5 expression is associated with more intense migration of thymic cancer cells, which can be prevented by silencing the gene (97). While all this substantiates the indispensable role of SCD5 in tumor development, it does not provide a clear answer to the question of whether desaturase plays a protective role against tumor malignancy or whether it should be considered a new therapeutic target (Table 1).

Given the reaction catalyzed by SCD5, its close relationship with carbohydrates and lipid metabolism is not surprising. Silencing SCD5 at protein and mRNA levels is closely correlated with a decrease in the number and size of lipid droplets, as well as in the level of PLIN2, a protein characteristic of these organelles (64, 97, 111). In a zebrafish animal model, *SCD5* gene knockout resulted in massive accumulation of visceral adipose tissue, among several other effects (58). SCD5 was found to be upregulated in human pancreatic islet cells in response to isoxazole treatment. Isoxazole is used as an antidiabetic drug, but it also affects cell differentiation, cardiac function and gene expression across a wide range (112). Increased SCD5 levels have also been described in nutrient deprivation (113) and shown to be associated with lysosomal lipid translocation (114) (Table 1).

The potential of SCD5 to influence diverse types of cell death appears to be substantiated by the findings, which collectively support its pivotal role in survival processes. In differentiated, weakly invasive, barely metastatic human breast carcinoma cells, SCD5 silencing induced uncontrolled, inflammation-associated necrosis-type cell death, which proved to be reversible by adding oleate (17). The role of SCD5 in modulating programmed cell death processes is also highly probable. In transgenic cell lines and in the *C. elegans* animal model, elevated SCD5 protein levels were found to correlate with reduced ROS production, likely as a consequence of increased MUFA synthesis, and with a reduction in apoptotic processes (114). Ferroptosis is a non-apoptotic form of programmed cell death that can be triggered by Fe-dependent lipid peroxidation. ASS1 plays an important role in resistance to ferroptosis by inducing SCD5 via SREBP1 TF (67) (Fig. 2A., Table 1), and thus silencing SCD5 sensitizes cells to this type of cell death (19). AutoPARylation of the PARP1 enzyme, which is characteristic of the induction of non-apoptotic programmed cell death parthanatos, has been observed in the absence of *SCD5* in gene-knockout primary glioblastoma stem-like cells. The loss of *SCD5* leads to an energetic crisis through NAD^+^ and ATP depletion, triggering PAR-mediated AIF nuclear translocation and parthanatos (22). Collectively, these findings support the pivotal role of SCD5 in survival processes, which may serve as a basis for a potential anti-tumor strategy.

## Contribution of SCD5 to embryogenesis

Lipid remodeling has been observed in several studies during the embryonic development of both invertebrates and vertebrates (115, 116, 117). A thorough investigation of the dynamic lipid landscape during early embryo development in mice and humans revealed that the high degree of phospholipid unsaturation is a conserved feature as embryos develop to the blastocyst stage. It is evident that lipid desaturases analogous to SCD1 are indispensable for in vitro blastocyst formation and implantation. A plausible mechanism underpinning this phenomenon involves the regulation of plasma membrane and apical protein fluidity facilitated by unsaturated FAs during the development of the eight-cell embryo into a blastocyst, as well as the establishment of apical-basal polarity in the same developmental stage (118). Given the exceptionally high expression of the *SCD5* gene in the gonad (48), it is plausible that this desaturase is responsible for the rearrangement of the PUFA-containing lipid profile observed during embryonic development, since SCD enzymes provide substrates for the biosynthesis of PUFAs and other complex lipids (46). In addition, a study conducted on chicken embryos and chicks has also shown that SCD5 expression exhibits a significant correlation with stearidonic acid (18:4 n-3) levels (119). However, this relationship is likely bidirectional, as while SCD5 may contribute to the shaping of the fatty acid profile, PUFAs may be able to inhibit the activity and transcription of SCD enzymes (120).

The potential role of SCD5 in optimal embryogenesis is reinforced by the fact that the *SCD5* gene was also identified as a candidate locus responsible for early embryonic mortality in pigs (121). Increasing oocyte nuclear maturity has been found to correlate with increasing *SCD5* expression in human cumulus cells. However, *SCD5* mRNA expression in oocytes capable of forming blastocysts by days 5–7 was significantly reduced in oocytes unable to develop beyond the embryo stage (60). The expression profile examined during the development of chicken embryos also exhibited a highly analogous temporal pattern (119). In addition, a comparison of mRNA sequencing data from cumulus cells of high-quality and low-quality embryos has revealed an association between poor embryo quality and elevated *SCD5* expression (109). The in vitro environment of the developing blastocyst can also trigger inadequate and unfavorable changes in desaturase levels (110). It is noteworthy that the indisputable role of SCD5 and unsaturated FAs can also be observed during spermiogenesis. In rabbit testicular tissue, a CLA-rich diet significantly increased *SCD5* mRNA expression, which in turn increased the expression of certain apoptotic genes (120). In consideration of the findings, augmented *SCD5* expression in oocytes and spermatozoa, followed by a sharp decline in expression subsequent to fertilization and the attainment of the few-cell blastocyst stage, may serve as a prerequisite for optimal embryogenesis, both in vitro and in vivo.

## Potential Relevance of SCD5 in human diseases

**Fig. 4:**
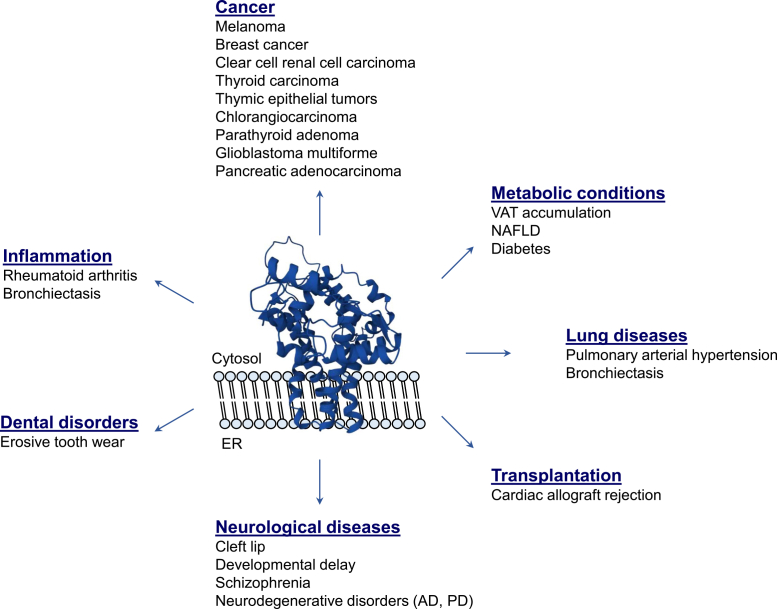
Summary of diseases connected to SCD5. The characteristics of the major disease categories associated with SCD5 are described in detail in the text.

Although the exact causal relationships and detailed molecular-level processes are still unclear in many cases, the pivotal role of SCD5 is convincingly emphasized by the fact that it is involved in the pathomechanism of numerous diseases affecting various systems and organs (Fig. 4).

### Neurological and neurodegenerative diseases

SCD5 is significantly expressed in both the adult and embryonic brain, at levels comparable to those of the SCD1 isoform (48). A complete absence of the *SCD5* gene and its chromosomal environment in humans may be associated with developmental anomalies and neurological symptoms. A pericentric inversion of chromosome 4, inv (4) (p13q21), has been identified with the inversion breakpoint positioned within the second exon of the *SCD5* gene, and its association with cleft lip has been confirmed in a two-generation family (43). The possible role of *SCD5* in palate formation has also been evidenced in another case of microdeletion 4q21 syndrome. This condition is originally associated with a combination of symptoms, including a broad forehead, widely spaced eyes, and prominent front teeth, as well as severe growth and developmental delay, and neonatal hypotonia, resulting from a deletion of a 1.37 MB region. However, the extended form, involving a 3.4 MB deletion that also affects the *SCD5* gene, was associated with cleft palate as an additional clinical feature (122). In addition to the inversion and deletion associated with complete SCD5 deficiency, a third chromosomal abnormality has also been described, in which the 4q21.21-q21.22 region, including the *SCD5* gene, is triplicated. Interestingly, this abnormality, presumably leading to greater *SCD5* expression as a result of de novo microtriplication, shares common features with the syndrome that develops in the absence of this chromosome region, e.g., developmental delay, muscular hypertension, and broad forehead, although it is supplemented by some new phenotypic features, i.e., relatively elongated extremities, a vascular malignant hemangioma in anamnesis, and elongated sigmoid colon (123).

The pathogenic role of an additional genetic anomaly of *SCD5* has also been suggested. A tryptophan-serine substitution at position 209 in the third transmembrane domain of the enzyme has been identified in a heterozygous form in non-syndromic, progressive sensorineural hearing loss in a five-generation Chinese family. The relevance of SCD5 protein is strongly supported by its notable expression in the inner and outer hair cells of the organ of Corti, the stria vascularis, cells of the lateral cochlear wall situated posterior to the spiral prominence, and most pronouncedly in spiral ganglion cells of guinea pig and human fetal cochleas. Furthermore, expression of the mutant SCD5 protein was also observed in the brain, which is consistent with the documented hearing loss feature (124).

Schizophrenia is a mental illness that affects 1% of the global population. The condition has a strong genetic basis, with an estimated heritability of 70%–80%, resulting from the complex interplay of numerous genes, each with a minor effect, in conjunction with environmental factors (125). In a genome-wide association study (GWAS) of considerable size, involving more than 50,000 SNPs, certain genes on chromosome 4, including *SCD5*, have been identified as susceptibility factors for schizophrenia (78). A base substitution within *SCD5* gene, affecting the binding site of the previously predicted and schizophrenia-related miR-20b miRNA in the 3′UTR, showed a very strong association with this mental condition (46, 78, 79).

Alzheimer's disease (AD) and Parkinson's disease (PD), the two most common age-associated neurodegenerative conditions, share similarities in gradual onset, progressive neuronal cell damage, and overlapping genetic risk factors; however, they differ significantly in their primary symptoms (126). *SCD1* and *SCD5* were identified as the most significantly altered genes in transcriptional profiling of primary neurons that expressed *α*-Synuclein (*α*Syn) with the amplified familial PD mutation 3K. Furthermore, desaturase inhibitors proved to be toxic in primary neurons, which could be prevented with oleate, the main product of SCD enzymes (127). Lipidomic analysis has shown that the amount of non-essential MUFAs is elevated in different brain regions, i.e., mid-frontal cortex, temporal cortex and hippocampus of AD patients. This is closely correlated with increased *SCD1*, *SCD5A*, and *SCD5B* transcription, and negatively correlated with cognitive function (128). Along these lines, advanced efforts are being made to develop SCD1-and/or SCD5-specific inhibitors, which may provide useful treatment for these neurological disorders (129). Regulation of *SCD5* expression may therefore be an important component of resilience to neurodegenerative conditions. The precise molecular mechanisms involved are largely unknown, but the retinoid X receptor (RXR) is likely to play a role. RXR regulates gene expression for a wide range of physiological processes, including development, metabolism, and cell differentiation. Its neuronally directed effects, such as neuron loss and impaired synapse integrity, have already been demonstrated in a mouse model of AD (130). Based on these findings, bexarotene, an RXR antagonist, is considered to have great therapeutic potential in AD and a number of other neurological disorders, such as stroke and PD. In an *α*Syn-induced PD model and in neurons derived from PD patients, bexarotene has been found to significantly reduce *SCD5* transcription as well as the size of developing lipid droplets (64).

### Metabolic conditions

Ectopic deposition of visceral adipose tissue (VAT) in the abdomen is usually accompanied by systematic metabolic dysfunction, an elevated risk of cardiovascular diseases, and T2DM. In zebrafish, it has been described that excessive VAT deposition develops in the *SCD5* homozygous mutant, accompanied by a shortened spine and abnormal body proportions. Based on RNA-seq results, this phenomenon can be attributed to the inhibition of canonical Wnt signaling and the significant activation of PPAR, C/EBP pathways, fat synthesis and fatty acid oxidation, and gluconeogenesis signaling in the scd5 −/− mutant, which was restored by *ω*-3 PUFA but not by stearate + palmitate or oleate + palmitoleate treatments (58). The key role of SCD5 in energy homeostasis is supported by the existence of miRNA binding sites in its 3′UTR, which can be linked to NAFLD and insulin resistance, considered to be a precursor of T2DM (46). While SCD5 downregulation associated with miR-484 was observed in the plasma of children with new-onset T2DM (90), the isoxazole antidiabetic drug increased *SCD5* expression in *β*-cells, protecting glucose-responsive signaling pathways under lipotoxic conditions (112). Furthermore, genetic variations in *SCD5* have been associated with both diabetes and NAFLD (59, 131) (see below).

### Cancer

In contrast to the ubiquitous upregulation of SCD1 in malignancies, emerging data suggest that increased *SCD5* expression often restrains tumor aggressiveness. For example, *SCD5* expression falls with disease progression in melanoma, and the re-expression of SCD5 (or addition of its product oleate) markedly reduces malignancy (14). Restoring SCD5 has been shown to acidify tumor cells and alter the secretion of extracellular matrix components and proteases (see Fig. 3), thereby inhibiting invasion (14). Furthermore, it has been demonstrated that SCD5 (or oleate) promotes differentiation in metastatic melanoma by inducing a partial mesenchymal-to-epithelial transition, increasing differentiation markers such as tyrosinase and melanin, and re-sensitizing cells to retinoic acid, resulting in a less aggressive phenotype (16). These findings support an anti-neoplastic role for SCD5 in melanoma (Table 2).

**Table 2:** Role of SCD5 in cancer

Role in Cancer	SCD5 Levels		Model System	Sample	Reference
	Protein	mRNA			
Increased melanoma malignancy	↓		cell culture	A375M, 4T1, Me1007	(14, 16)
Decreased melanoma progression	↑		cell culture	A375M, 4T1	(14)
Increased metastasis formation in melanoma	↓	↓	cell culture	A375M, Me1007	(16)
Increased ATRA sensitivity of melanoma	↑		cell culture	A375M	(16)
Forced mesenchymal to epithelial transition in melanoma	↑		cell culture	A375M	(16)
Neoadjuvant chemotherapy for stage II/III breast cancer, responder		↓	high-throughput	breast cancer biopsy	(153)
Increased necrosis in breast carcinoma	↓	↓	cell culture	MCF-7, MDA-MB-231	(17)
Breast cancer	↑		cell culture	MCF-7 and 10A, SK-BR-3, MDA-MB-231 and 316	(133)
Increased breast cancer malignancy		↓	high-throughput	human breast cancer biopsy samples	(132)
Longer disease or relapse free survival in breast cancer		↑	high-throughput	human breast cancer biopsy samples	(132)
Decreased breast cancer malignancy, metastasis, tumor size	↑	↑	animal model	BALB/cAnNCrl mice	(18)
Increased mesenchymal to epithelial transition in triple negative breast cancer	↑	↑	cell culture	4T1	(18)
Increased TNBC overall- and relapse free survival		↑	high-throughput	human TNBC samples	(18)
Decreased risk of clear cell renal cell carcinoma		↓	high-throughput	human ccRCC renal tissue sample	(152)
Increased risk of clear cell renal cell carcinoma		↓	high-throughput	human ccRCC renal tissue sample	(135)
High risk of clear cell renal cell carcinoma		↓	high-throughput	human ccRCC renal tissue sample	(134)
Decreased overall survival in clear cell renal cell carcinoma		↓	high-throughput	human ccRCC renal tissue sample	(69)
Unfavorable uveal melanoma prognosis		↓	high-throughput	human primary and metastatic UVM cells	(136)
Increased overall survival in intrahepatic cholangiocarcinoma		↑	high-throughput	human IHCC samples	(77)
Unfavorable prognosis of thymic epithelial tumor		↑	high-throughput	human thymoma	(97)
Diagnosis of anaplastic thyroid cancer		↑	high-throughput	anaplastic thyroid carcinoma	(154)
Diagnosis of pancreatic adenocarcinoma		↓	high-throughput	primary tumors of PC	(137)
Increased size of parathyroid adenoma		↓	high-throughput	human PA samples	(138)
Decreased glioblastoma stem cell survival	↓	↓	cell culture	primary glioblastoma stem-like cells	(22)

SCD5 also appears to have a protective effect in breast carcinoma. Bioinformatic analyses reveal that *SCD5* mRNA is often downregulated in aggressive tumors, and low *SCD5* levels are associated with poor prognosis and poor response to neoadjuvant chemotherapy (132). *In vitro*, tumor stroma can upregulate *SCD5*, as cancer-associated fibroblasts have been shown to induce SCD5 in MCF7 cells to promote survival. However, *SCD5* knockdown triggers tumor cell necrosis, which can be prevented by oleic acid (17). Conversely, enforced expression of SCD5 in a triple-negative breast cancer (TNBC) model reduced metastatic spread by blocking SPARC secretion and reversing EMT (18). SCD5-overexpressing tumors also exhibited reduced myeloid immunosuppression and increased T-cell activation via an oleate/SPARC-dependent mechanism (18). Consistent with these findings, SCD5 (along with other lipogenic enzymes) is elevated in breast cancer cell lines and correlates with higher saturated/monounsaturated lipid levels (133) (Table 2).

*SCD5* is often suppressed in kidney cancer. In clear cell renal cell carcinoma, the loss of the VHL tumor suppressor or hypoxia-inducible factors downregulate *SCD5* via HIF signaling (see Fig. 2A). Conversely, reduced SCD5 shifts cellular lipidomics and promotes proliferation, whereas higher SCD5 expression in tumors is associated with longer survival (69). Accordingly, SCD5 has been identified as a contributing factor in computational prognostic signatures for renal cancer (134, 135). For instance, *SCD5* was identified among a seven-gene risk model for ccRCC (134). Similarly, *SCD5* was incorporated within a mitophagy-related ccRCC prognostic signature (135) (Table 2).

Beyond melanoma, breast, and kidney cancers, alterations in *SCD5* expression have been noted in a range of other cancers. In uveal melanoma, SCD5 has been identified as an independent prognostic factor associated with metastasis (136). Intrahepatic cholangiocarcinoma signatures include *SCD5* among key metabolic prognostic genes (77). In thymic carcinoma, an oncogenic lncRNA (LINC00174) has been shown to sponge miRNA-145-5p, thereby upregulating *SCD5* and promoting tumor cell migration and changes in lipid metabolism (97) (Fig. 2B). However, pancreatic ductal adenocarcinoma exhibits consistent downregulation of *SCD5* (and other PPAR-pathway genes) compared to normal tissue, particularly in KRAS-mutant cases (137). In a proteomic study of parathyroid adenomas, higher *SCD5* levels were found to be inversely correlated with tumor size (138), suggesting that SCD5 may have a role in the regulation of growth (Table 2).

It is noteworthy that in glioblastoma (GBM) stem-like cells, SCD5 plays a pivotal role in sustaining proliferation and DNA repair. SCD5 is highly expressed in GBM cells, and its knockdown, often paired with SCD1, has been observed to cause SFA accumulation, which in turn has been shown to hyperactivate and then degrade PARP1. This results in the depletion of RAD51 and subsequent impairment of homologous recombination, leading to the triggering of a PARP-mediated form of cell death known as parthanatos. *In vivo*, SCD5 depletion has been shown to significantly extend survival in GBM models, thereby revealing a lipid-mediated vulnerability of tumor DNA repair (22) (Table 2).

Collectively, these studies portray SCD5 as a context-dependent regulator of tumor metabolism and microenvironment. In many cancers (melanoma, breast, renal, etc.), SCD5 expression or oleate production supports differentiation and epithelial phenotypes, limits EMT and metastasis, and promotes anti-tumor immunity (14, 18). On the other hand, loss or downregulation of SCD5 is a recurrent feature of advanced tumors and generally associates with poorer outcome (14, 132).

### Other health conditions

Rheumatoid arthritis (RA) is a common and disabling chronic inflammatory disease with an extremely complex pathogenesis. Examining the miRNA expression profile of T-cells in RA patients, revealed increased levels of miR-34b, which may target the 3′UTR of *SCD5*, thereby reducing its expression (87). Bronchiectasis, another chronic inflammatory disease characterized by unusual enlargement and destruction of the bronchial tubes and recurrent infections, also correlates with *SCD5*. A large-scale genotype-phenotype correlation study has revealed that increased SCD5 expression is associated with a lower risk of this lung disease (139). *SCD5* overexpression has also been detected in connection with another lung disease, pulmonary arterial hypertension (PAH). Increased *SCD5* mRNA levels were detected in peripheral blood samples from vasodilator-responsive PAH patients (140).

In addition to its role in inflammatory processes, SCD5 may also influence the development and modulation of the immune response. Consequently, its level could potentially serve as a biomarker for predicting cardiac allograft rejection (141). Furthermore, it may also be a genetic component in erosive tooth wear, although this is not yet clear (142).

## Methodological Approaches in the Research of SCD5 function

### High-throughput screenings

Over the past two decades, a gradual accumulation of experimental data has revealed that SCD5 plays a highly significant role in cell metabolism, proliferation, and differentiation. However, the discovery that desaturases are involved in such fundamental processes in the cells has actually increased, rather than dispelled the uncertainty surrounding the exact function of these enzymes. This is probably why, and of course due to methodological advances, there is an increasing number of large-scale *SCD5* mRNA expression analyses being performed (Supplemental Table S3). In addition to human samples, increasing phenotypic diversity is being compared with mRNA profiles in more and more species, and significant changes in *SCD5* expression are frequently observed. High-throughput mRNA, exome or genome sequencing results are also analyzed using bioinformatic methods. However, with a few exceptions that are highly informative in terms of function, changes in the *SCD5* mRNA profile are not followed up at the molecular level, and there is no attempt to map the precise intracellular mechanisms. In line with this, a growing number of studies draw conclusions from further analysis of previous expression profiles found in databases rather than from freshly generated experimental data (Supplemental Table S3).

Based on the established role of SCD1 isoform as a master regulator of lipid metabolism, numerous changes in *SCD5* mRNA expression profile have been described in response to various treatments, diets, or conditions that are partially or wholly related to lipid homeostasis (Supplemental Table S3). The vast majority of these are non-human and are based on RNA samples prepared by the given research group. They primarily target the molecular background of factors relevant to animal husbandry, such as body weight, milk yield, and reproduction. Significant reductions in *SCD5* mRNA expression have been described in response to starvation in scallop hepatopancreas (113), in vitro produced cattle blastocytes (110), and following acorn and degradable starch diets in pig dorsal subcutaneous fat (143) and goat mammary tissue (144), respectively. Interestingly, environmental stress, in addition to nutrient stress, also resulted in decreased *SCD5* expression in mud crab hepatopancreas exposed to high temperatures and strong illumination (145). In contrast, increased *SCD5* mRNA expression was measured in cattle intramuscular fat (146, 147), chicken preadipocytes (148) and calf muscle tissue exposed to guanidinoaceitic acid and methionine-containing diets (149), as well as creep feeding (150). To date, only one database-initiated study has been conducted on samples from non-human vertebrates, which supports altered *SCD5* mRNA expression in cattle mammary tissue (151).

It is worth noting that, unlike SCD1, the relevance of SCD5 in lipid metabolism remains elusive, as only a few large-scale analyses have found a correlation with human SCD5 in this domain. Reduced *SCD5* mRNA expression appears to correlate with smaller lipid droplets in both human thymic carcinoma and monocyte cell lines (97, 111) and may influence the pathophysiology of T2DM (90). At the same time, isoxazole, an antidiabetic agent, has been linked to higher *SCD5* levels (112).

Although the effect of SCD5 on lipid composition is undeniable, its role in this regard appears to be increasingly overshadowed by that of SCD1. On the other hand, the enzyme is receiving increasing attention in cancer research, as it appears on the lists of differentially expressed genes (DEGs) in connection with an increasing number of tumor types, and its altered expression can be demonstrated in large-scale studies (Supplemental Table S3). Significantly lower levels of *SCD5* have been demonstrated in ccRCC (69, 134, 135, 152), breast cancer (18, 132, 153), thymic epithelial tumors (97), intrahepatic cholangiocarcinoma (77) and pancreatic adenocarcinoma (137). However, with one exception (153), these studies are based on transcriptomic datasets of primary cancers and matched normal samples collected as part of The Cancer Genome Atlas (TCGA) program or re-evaluations of sequencing results from the METABOLIC, ICGC, PTAC or GEO databases (Supplemental Table S3), so similar expression shifts are not surprising. Nevertheless, significant *SCD5* mRNA upregulation has been measured both in freshly collected melanoma samples (22) and in the analysis of previous transcriptomic data from the GEO database (136), similar to the anaplastic thyroid carcinoma samples (154).

In addition to metabolic and tumor diseases, significant results for *SCD5* were also found when analyzing the mRNA expression profiles of several other unrelated or only loosely related conditions. These RNA-seq results, obtained from the original sample set corresponding to the experimental setup of the given working group, suggest a consistent increase in *SCD5* expression in various conditions, including AD (128), bronchiectasis (139), poor embryo quality (109), WWOX deficiency (20), ASS1 overexpression (67) and pulmonary arterial hypertension (140). However, in cases of cardiac allograft rejection, a decrease in *SCD5* mRNA levels was identified in peripheral blood samples analyzed from the GEO database as a possible biomarker (141) (Supplemental Table S3).

Currently, the volume of high-throughput data is increasing, yet different *SCD5* expression profiles are often described for the same disease. Thus, the only conclusion that can reliably be drawn at present is that SCD5 is involved in changes in nutrient supply or environmental stress, as well as in tumor processes, and that its expression changes accordingly. However, due to the small number of molecular-level studies, it cannot be clearly stated whether changes in SCD5 levels are the cause or consequence of these processes.

### Genetic variations of SCD5 gene

Although large-scale transcriptomic data can contribute significantly to the exploration of the diverse functions of SCD5, they cannot replace targeted experimental work that focuses on investigating the role of a few selected genetic variants. This is because the specific effects and consequences of individual genetic variations in the enzyme or their close correlation with certain phenotypic traits further refine our understanding of the role of SCD5. Alongside the influence of SCD1 on lipid homeostasis, *SCD5* mutations and polymorphisms are also frequently studied to identify markers relevant to livestock breeding parameters (Table 3). For instance, *SCD5* was identified as a genetic marker for pig growth rate in an association study using a porcine SNP database (155). Marker-assisted breeding is also considered to be of great importance in cattle. Marker-trait association studies are used to identify genetic loci that could facilitate genetic and metabolic selection in cattle. *SCD5* polymorphisms proved to be highly representative of milk SFA/UFA levels, as the rs252452204 SNP G allele was correlated with a reduced SFA/UFA ratio and increased MUFA and CLA-11 content, while the rs252452202 T allele showed the opposite, resulting in increased SFA and lower MUFA content in milk (156). A third variant (rs43687643) T allele was associated with decreased SFA and increased PUFA levels (156). The latter was also confirmed in grass-fed beef, where the desaturation index for 16-carbon FAs was found to be higher in animals with the rs43687643 T allele (157). In addition to SNPs, larger-scale polymorphisms were also studied in Holstein bulls, and CNVs involving the *SCD5* gene have been identified in relation to milk composition (158). A GWAS revealed the influence of *SCD5* gene-related SNPs on cow's cheese fragrance as a contributor to the distinctive flavor of cheese (159). In addition to cattle, research into genetic variants in sheep is also advancing. Several GWAS have been conducted to identify the genetic components of phenotypic traits related to meat yield in sheep, and all found that *SCD5* SNPs are associated with body size (160), chest width and lamb size (161), and meat productivity traits (162).

**Table 3:** Analyzed genetic variations of human SCD5

SNP ID	Position	Nt Change	Effect	Population	Method	Phenotype	Correlation	Reference
*rs192654796*	exon	G > C	p.W209S	Chinese	WES, pedigree cell culture	risk for non-syndromic deafness proliferation	increased decreased	(124)
*rs138660783*	exon	T > G	p.F151C	European	WGS, AA	risk for hearing loss	increased	(163)
*rs3733228*	exon	C > A	p.L246M	Chinese	WGS, AA	risk for NAFLD	increased	(131)
*rs1358056*	intron	A > G	‒	Chinese	GWAS	risk for schizophrenia	increased	(78)
*rs7682394*	intron	T > G	‒	mixed	GTEx	expression in VAT	decreased	(164)
*rs145164872 rs1430176385 rs140750150 rs1250613148 rs1225904796 rs1011850309*	splice sites	A > G G > A C > T,G C > A C > T G > C,A	altered splicing site	‒	cell culture	altered SCD5A/SCD5B ratio	‒ increased ‒ ‒ ‒ decreased	(48)
*rs10033464*	5′ flanking	GT → T	‒	Finnish	GWAS	risk for erosive tooth wear	increased	(142)
*rs3811792*	promoter	T > C	TF binding site modification	Caucasian	cell culture AA	promoter activity risk for diabetes	decreased increase	(59)
*rs10007189 rs17006523*	3′ flanking	C > T G > A	‒	mixed	GTEx	expression in hypothalamus	decreased	(164)
*rs6840 rs1065403 rs3821974 rs373323*	3′UTR	C > T A > G G > A C > T	‒	Korean	AA	risk for hepatocellular carcinoma	decreased	(165)

Nevertheless, association and functional studies of genetic variations in the human *SCD5* gene are also emerging, which may greatly contribute to mapping the pleiotropic functions of the enzyme (Table 3). Pedigree analysis and whole-exome sequencing identified a missense *SCD5* variant (rs192654796) that causes non-syndromic, progressive sensorineural hearing loss in an autosomal dominant inheritance pattern in a five-generation Chinese family. This substitution of tryptophan for serine at amino acid position 209 in the third transmembrane domain of SCD5 transcript variant A reduced proliferation in a cellular model (124). Remarkably, another amino acid change affecting the coding region (rs138660783, p. F151C) has been associated with enlarged vestibular aqueduct and subsequent hearing loss. The mutation has not yet been functionally characterized, but its pathogenic effect has been predicted in silico (163). A third missense polymorphism (rs3733228, p.L246M) located near the C-terminus of the SCD5 transcript variant B, interacts negatively with two other genetic variations associated with NAFLD in obese children (131).

To date, two intronic variants of *SCD5* have been studied. The rs1358056 was found to increase the risk of schizophrenia in the Chinese population (78), while the rs7682394 variant was found to be associated with reduced *SCD5* expression in visceral adipose tissue (164). The two *SCD5* transcript variants are produced by unequal splicing via an alternative terminator, where the probability of the process yielding SCD5A is an order of magnitude higher. Investigating donor and acceptor splice site mutations involved in alternative splicing identified two variants that modify the SCD5A/SCD5B ratio at both the mRNA and protein levels. While rs1430176385 reduced the already low SCD5B expression, rs1011850309 shifted the ratio towards the SCD5B transcript variant by reducing SCD5A levels (48).

Of the numerous genetic variations found in the 5′ regulatory region of *SCD5*, one (rs10033464) showed a significant association with erosive tooth wear, interestingly only in women (142). The minor allele of the rs3811792 SNP, which is located in the promoter, was also significantly associated with T2DM and T1DM, and proved to be functionally significant, as it decreased transcriptional activity in an in vitro luciferase reporter system, and reduced the binding probability of the ETS1 transcription factor in silico (59). Based on data from the Genotype-Tissue Expression project, the minor alleles of two SNPs (rs10007189 and rs17006523) located in the 3′ flanking region of *SCD5*, which otherwise has high brain expression, are significantly correlated with downregulation of the enzyme in the hypothalamus (164). At the same time, in hepatocellular carcinoma with reduced *α*-fetoprotein levels, an enrichment of the minor alleles of four SNPs (rs6840, rs1065403, rs3821974, and rs373323) affecting the 3′UTR of *SCD5A* was observed in the Korean population (165).

## Conclusion

SCD5 is a context-dependent, tissue-specific *Δ*9-desaturase that appears to serve functions distinct from the broadly regulated lipogenic enzyme SCD1. Its expression is evolutionarily conserved in non-rodent vertebrates and is mainly seen in neural, pancreatic, and certain reproductive tissues, suggesting a role in local lipid remodeling rather than bulk energy storage. SCD5 alters the intracellular MUFA/SFA balance and thereby affects membrane composition, vesicular trafficking, receptor signaling, and Wnt protein processing. In this way, it emerges as a regulator of signal transduction and cell fate, not merely as a supplier of triglyceride precursors.

SCD5 also shows strong context dependency in proliferation–differentiation balance and in tumor biology, where it may act either as a differentiation-promoting or survival-supporting factor depending on the cellular setting. Its regulation is unusually complex, involving alternative splicing, promoter and 3′UTR variants, miRNAs, and transcription factors linked to development, hypoxia, and metabolic stress.

Although the post-translational control and protein stability of SCD5 have not yet been fully characterized, it has become evident that they differ from those of SCD1. The N-terminus of SCD5 lacks the canonical PEST motifs present in SCD1. Variable half-lives have been reported for SCD5 across different cell types and tumor stages, with both proteasomal and non-proteasomal degradation contributing to this variability. This distinctive stability profile is likely to contribute to cell-type-specific SCD5 protein dynamics, emphasizing the necessity for direct biochemical characterization of potential sites of ubiquitination and other post-translational modifications.

Beyond all this, it should not be forgotten that genetic variants in the human *SCD5* gene remain largely understudied, even though they may influence every aspect of the enzyme’s function as it ultimately manifests within the cell. According to the NCBI database, of the nearly 2,000 SNPs currently found in the gene, only 19 have been studied functionally or at the population level to date (Table 3), so relatively little is known about the consequences of individual differences in SCD5, which, however, cannot be overlooked in the era of personalized medicine.

Despite the accumulation of mechanistic and correlative data, critical knowledge gaps remain regarding the SCD5 enzyme and its role. The currently inconclusive substrate preference spectrum, i.e., the relative activity of the enzyme toward C16 versus C18 acyl-CoAs under physiological conditions, still needs to be elucidated. The approach to the issue of functional redundancy versus specialization relative to other *Δ*9-desaturases depends on the tissue and experimental model. In addition, the absence of an SCD5 ortholog in rodents complicates in vivo modeling and increases reliance on alternative vertebrate systems and engineered humanized models.

In summary, SCD5 has specialized functions in lipid remodeling, integrally linked to membrane-dependent signaling, developmental programs, and context-specific stress responses. Elucidation of its enzymology, regulatory mechanisms, and tissue-specific roles is imperative to comprehend the contribution of MUFA production by SCD5 to physiological processes such as neurodevelopment, islet biology, and reproduction, as well as pathological states such as tumor progression, metabolic dysregulation, and susceptibility to ferroptosis. SCD5 is emerging as a promising drug target in a variety of diseases. However, due to its implication in a number of diverse context-dependent actions, the development of effective therapeutic strategies targeting SCD5 protein will require the implementation of precise methodologies that are tailored to the specificities of the tissue concerned, as well as to the disease state and the underlying regulatory milieu.

## Data Availability

Not applicable.

## Supplemental data

This article contains supplemental data.

## Conflict of interest

The authors declare that they have no conflicts of interest with the contents of this article.
