# Supplementary material for: Stearoyl-CoA desaturase 5 (SCD5) in lipid remodeling: From molecular control to pathophysiology
Source: J Lipid Res. 2026 Jun 12;67(7):101078. doi: 10.1016/j.jlr.2026.101078 (PMC13377153; doi:10.1016/j.jlr.2026.101078)
Supplement: Supplemental Tables [file mmc1.pdf]

SUPPLEMENTARY INFORMATION

for

Stearoyl-CoA Desaturase 5 (SCD5) in Lipid Remodeling: From Molecular Control to Pathophysiology

Veronika Zámbó, Gabriella Orosz, Miklós Csala, Éva Keresztúri*

Department of Molecular Biology, Semmelweis University, H-1085 Budapest, Hungary

* for correspondence: Éva Keresztúri; Department of Molecular Biology, Semmelweis University, H-1085, Budapest, Hungary; kereszturi.eva@semmelweis.hu

Short title: SCD5: Mechanisms and Diseases

Table S1. ***Hypothetical but experimentally unconfirmed transcription factors in the SCD5 promoter.***

**Transcription factors**	**Species**	***In silico* prediction**	**Correlation with SCD5 expression**	**Reference**
C/EBPa, AP1, SP1, NF-Y, NF-1, SREBP1, PPARa, T3R	human	yes	–	(46)
MITF, ZEB2	human	no	positive	(16)
ZEB1, TWIST, SLUG	human	no	negative	(16)
SPI1, MEIS3, SOX18, NFIA, TFE3, TFAP2A, SOX10, SOX2, MEIS2, RBPJ, NFATC2, ZNF354C, NFATC3, MZF1, ETS1	human	yes	–	(59)
Sp1, AP-1, NFR-1, ACE2, AP-2a, C/EBPb, E4, GR, MRF4, NF-1, Oct-1	chicken	yes	–	(63)
RXR	human	no	negative	(64)

Table S2. ***In silico predicted but experimentally unconfirmed miRNAs involved in the regulation of SCD5 expression.***

**miRNAs**	**Prediction tool**	**Susceptibility to disease**	**Relation to cancer**	**Reference**
miR-106a	TargetScan	cancer, autism	yes	(46,80)
miR-20b	TargetScan	cancer, schizophrenia	yes	(46,78,79)
miR-17	TargetScan	cancer, glioma	yes	(46,81)
miR-205, miR-221, miR-222, miR-17-5p, miR-20a	TargetScan	pancreatic cancer	yes	(46)
miR-200ab, miR-17	TargetScan	NAFLD	no	(46)
miR-1928	TargetScan	PTSD	no	(82)
miR-141, miR-93, miR-93a, miR-105, miR-291a-3p, miR-294, miR-295, miR-302abcde, miR-20b-5p, miR-106ab, miR-427, miR-518a-3p, miR-411, miR-494, miR-205ab	TargetScan	various types of cancer	yes	(46)
miR-34b	TargetScan	rheumatoid arthritis	no	(87)
miR-27a-3p, miR-27b-3p	StarBase	cholangiocarcinoma	yes	(77)
miR-484	TargetScan, Miranda	T2DM	no	(90)

Table S3. ***High-throughput analysis of SCD5 mRNA profiles associated with diseases and intracellular processes.***

Disease, phenomenon	Change in SCD5 mRNA expression	Species	Dataset source	Reference
Degradable starch diet	↓	goat	own experimental data	(144)
Acorn-based diet	↓	pig	own experimental data	(143)
Starvation	↓	scallop	own experimental data	(113)
In vitro produced blastocytes	↓	cattle	own experimental data	(110)
High temperature, high illumination	↓	mud crab	own experimental data	(145)
Intramuscular fat	↑	cattle	own experimental data	(146,147)
Preadipocyte	↑	chicken	own experimental data	(148)
Guanidinoacetic acid and methionine rich diet	↑	cattle	own experimental data	(149)
Creep diet	↑	calf	own experimental data	(150)
Mammary tissue	↑	cattle	database (UniGene, ESTProfile)	(151)
Lipid droplet size reduction	↓	human	database (TCGA, IRE, GEO)	(97)
Lipid droplet size reduction	↓	human	own experimental data	(111)
T2DM	↓	human	own experimental data	(90)
Isoxasole	↑	human	own experimental data	(112)
Clear cell renal cell carcinoma	↓	human	database (TCGA)	(134)
Clear cell renal cell carcinoma	↓	human	database (TCGA, GSEA)	(69)
Clear cell renal cell carcinoma	↓	human	database (TCGA, HPA)	(135)
Clear cell renal cell carcinoma	↓	human	database (TCGA, CPTAC, ICGC, EMBL)	(152)

Continuation of Table S3.

**Disease, phenomenon**	**Change in SCD5 mRNA expression**	**Species**	**Dataset source**	**Reference**
Breast cancer	↓	human	own experimental data	(153)
Breast cancer	↓	human	database (GTEx, TCGA, GEO)	(132)
Triple-negative breast cancer	↓	human	database (METABRIC)	(18)
Thymic epithelial tumor	↓	human	database (TCGA, IRE, GEO)	(97)
Intrahepatic cholangiocarcinoma	↓	human	databasel (TGCA, GEO)	(77)
Pancreatic adenocarcinoma	↓	human	database (TGCA)	(137)
Melanoma stem cell	↑	human	own experimental data	(22)
Poor uveal melanoma prognosis	↑	human	database (GEO)	(136)
Anaplastic thyroid carcinoma	↑	human	own experimental data	(154)
Cardiac allograft rejection	↓	human	database (GEO)	(141)
AD patientes	↑	human	own experimental data	(128)
Bronchiectasis	↑	human	own experimental data	(139)
Low embryo quality	↑	human	own experimental data	(109)
WWOX deficiency	↑	human	own experimental data	(20)
Pulmonary arterial hypertension	↑	human	own experimental data	(140)
ASS1 overexpression	↑	human	own experimental data	(67)
